# Comments on Jo and Colleagues' Paper (2010) "Association of Subway Driver's Depressive Symptoms and Experience of Work-Related Problems"

**DOI:** 10.4178/epih/e2011002

**Published:** 2011-04-25

**Authors:** Jae-Min Kim

**Affiliations:** Department of Psychiatry, Chonnam National University Medical School, Gwangju, Korea.

## Background

Certain occupations are at greater risk of stress and depression than others. In particular, occupations related to too much or too little work, having to make too many decisions, having very little control over decisions, time pressures and deadlines, excessive and inconvenient working hours, highly repetitive work and lack of job variety, the necessity to work fast, job insecurity and the prospect of redundancy or being forced into premature retirement have all been reported to contribute to stress and depression [1,2]. Subway drivers are particularly exposed to these risk factors. Work stress and depression can also exacerbate employees' chronic illness and, in effect, be counterproductive in terms of working ability and outcomes [3]. As the number of subway users increases, subway drivers' psychological problems are important not only on a personal level but also on a public health one.

## Why was this study performed?

More than 16 billion people a year use subway trains in Seoul, the city ranked third behind Tokyo and Moscow. Unfortunately, there are no available data in this respect in Korea, being the similar condition in other particular occupational categories. Mental health problems have actually been neglected. Therefore, there is an urgent need to investigate the work-related stress and psychological problems of subway drivers in Korea. Jo and colleagues' recently published article "Association of subway drivers' depressive symptoms and experience of work-related problems" is certainly well timed [4].

## What did the researchers do and find?

The researchers recruited 827 subway drivers in Seoul. Each participant was assessed with a survey protocol on socio-demographic and common occupational characteristics. Information on work-related problems and stress were obtained by self-report in four sub-domains: an accident resulting in death or injury, a conflict with a customer, a sudden stop in response to an emergency bell, and a near accident. Depression was categorized using a cut-off point of 21 on the Center for Epidemiologic Studies-Depression (CES-D) Scale [5,6]. All data were gathered at the same time point, and cross-sectional associations between four work-related problems and depression were examined by univariate (χ^2^ tests) and multivariate (logistic regression tests) analyses. The researchers found that a sudden stop in response to an emergency bell was independently associated with depression. The association between near accident and depression showed borderline significance.

## What do these findings mean?

This study is valuable because of its rarity. The survey on work-related problems and depression of Korean subway drivers is an end in itself. Furthermore, these findings provide additional support for the idea that increased levels of work problems and stress are associated with depression in subway drivers. Because the design of this study was cross-sectional, however, the causal pathway between work problems and depression could not be determined. That is, work problems and stress may increase depression in subway drivers as claimed by the authors, and in turn drivers with depression can experience work-related problems more frequently compared with those without depression. There is strong evidence that depression and psychological problems cause work-related errors and accidents [7]. Further prospective study is needed to clarify the causal relationship between work problems and depression in subway drivers. Overall, this study suggests an interrelationship between work-related problems and depression, and therefore intervention to prevent one of these could reduce the risk of the other. There is evidence that supervisor support, as one of the dimensions of workplace support, has a beneficial effect on health outcomes among employees, particularly for depression [8,9]. It has also been found that social support at work is directly related to high job control, low depression and high job performance [10]. These issues also need to be pursued in future research.
